# Towards the design of anti-amyloid short peptide helices

**DOI:** 10.6026/97320630014001

**Published:** 2018-01-31

**Authors:** Irena Roterman, Mateusz Banach, Leszek Konieczny

**Affiliations:** 1 Department of Bioinformatics and Telemedicine, Jagiellonian University-Medical College, Lazarza 16, 31-530 Krakow, Poland; 2 Chair of Medical Biochemistry, Jagiellonian University-Medical College, Kopernika 7, 31-034 Krakow, Poland

**Keywords:** Amyloid, drug design, hydrophobicity

## Abstract

A set of short peptide sequences susceptible to fibrillar aggregation produces sequneces capable of arresting elongation of amyloid fibrils. The "stop" signals are short helices customized for each individual target. Such a helix should exhibit high amphiphilicity, with differing conditions present on each side (one side should be highly hydrophilic to enable water to interact with the aggregate, while the other side must retain a local distribution of hydrophobicity which matches that of the terminal portion of the fibril). The emergence and elongation of fibrillary forms resulting from linear propagation of local hydrophobicity peaks is shown using the fuzzy oil drop model.

## Background

An important voice in the discussion regarding Alzheimer's disease comes from the psychologist community, which attempts to identify objective causes for the disase [1]. Clinicians tend to base their diagnoses on the pathological amassment of amyloid-β (Aβ) plaques in the central nervous system [2]. The involvement of gangliosides and cholesterol in forming amyloids is based on a common mechanism [3], leading to the conclusion that a universal therapeutic process targeting neurodegenerative diseases may be devised. A comprehensive discussion of the so-called Energetic Funnel pathway, founded upon thermodynamic principles, which likens the folding process to the search for an internal energy minimum, of the role of chaperones and chaperonins in the folding process and of the relation between the structural stability of proteins and pathological processes implicated in misfolding diseases is discussed elsewhere [4]. The work also highlights therapeutic options - such as therapeutic inhibition of precursor protein synthesis through expanding the use of RNA interference (RNAi). Other notable approaches to drug discovery include research into chaperone expression and vaccines [5]. Nevertheless, the most promising avenue of research appears to involve short synthetic peptides containing the self-recognition motif of the protein and engineered to destabilize the abnormal conformation, which might be useful to correct protein misfolding [6]. Such peptides are sometimes referred to as minichaperones. They exhibit affinity for areas responsible for selfassociation and contain residues that specifically favor or disfavor a particular structural motif. The use of polyphenols is reported elsewhere [7] on the basis of hydrophobic arguments. Enzymes capable of breaking up amyloids [8], study the possible applications of nanoparticles in misfolding disease treatment [9] or investigate the properties of cyclic cis-locked phosphor-dipeptides are also known [10]. Physical methods include femtosecond laser-induced nanoexplosion of gold nanorods [11], while clearance and degradation of amyloid β peptides was observed with the use of anti-inflammatory Annexin A1 [12]. It was further noted that aducanumab (a human monoclonal antiboty) reduces Aβ plaques in Alzheimer's disease [13]. The search for novel drugs is not, however, based on amyloid plaque formation mechanisms. According to the fuzzy oil drop model [14] (FOD) the water environment plays a decisive role in this process - as indeed noted by other authors who recognize the importance of hydrophobic interactions for amyloid formation. Research into structural properties of water is carried out on both theoretical [15] and experimental grounds [16]. The external environment is also recognized as a factor in amyloidogenesis [17].

The fuzzy oil drop model describes the existence of a hydrophobic core, with hydrophobic residues congregating at the center of the molecule and hydrophilic residues exposed on the surface. This type of structure favors interaction with water [14], while any local deviations from the theoretical distribution of hydrophobicity (mathematically expressed by a 3D Gaussian) are suspected of mediating biological activity. More specifically, local hydrophobicity deficiencies usually correspond to ligand binding sites [18], while areas of excess hydrophobicity, if present on the surface, may indicate complexation sites for other proteins with similar characteristics [19]. Such local discordances are likened to the "iceberg" model were means of communication between molecules in water is discussed [20].

The fuzzy oil drop model also reveals another type of discordance versus the theoretical monocentric hydrophobic core: linear propagation of local hydrophobicity peaks interspersed by local minima. This situation occurs when the polypeptide (or polypeptides) is unable to fold as an individual molecule with a monocentric FOD-compliant hydrophobic core, and instead folds in a way, which is dependent only on the intrinsic hydrophobicity of each individual residue. If the resulting fragment is placed in the proximity of other similarly folded fragments, linear propagation becomes highly likely. As shown in [21], once linear propagation sets in, a "stop" signal is needed to halt it [22]. Such stop signals have indeed been identified in the structures of many proteins whose native forms comprise elongated cylinder-like fragments, e.g. solenoids. Clearly, evolution has devised ways to prevent unchecked linear propagation of protein chains. On the basis of this observation we have designed several peptides that serves as "stoppers" for the amyloid-forming chains are listed [23].

## Methodology

### Dataset

We base our study on the set of amyloid-forming popetides discussed in [23] and treated as targets for the design of stoppers, which would prevent elongation of amyloid fibrils. The target proteins are listed in Table 1.

The pattern for the design of a "stop" signal is provided by a lyase - bacterial chondroitinase b pectate lyase (PDB ID: 1DBG) [32]. This protein contains a solenoid fragment with a notably linear arrangement of hydrophobic and hydrophilic "bands". This type of structure might propagate indefinitely in the absence of an amphiphilic helical stopper, whose hydrophilic side faces the water environment while the hydrophobic side remains in contact with the fibril. Thus, the protein does not readily form complexes or grow indefinitely.

### Fuzzy oil drop model

The fuzzy oil drop model has been described in detail elsewhere [33, 34]. According to the model, the theoretical distribution of hydrophobicity in a protein body can be modeled by a 3D Gaussian, which peaks at the center of the encapsulating ellipsoid. Proteins that conform to this model with good accuracy include titin [35] as well as antifreeze class II [36] and downhill proteins [37]. An in-depth study of all domains present in the PDB database (nonredundant set [38]) revealed that the vast majority of individual domains in a way which ensures compliance with the theoretical Gaussian distribution. This does not, however, rule out the presence of discordances and deviations - indeed, local departures from the theoretical hydrophobicity distribution often correspond to ligand binding sites [18] or complexation sites, capable of attracting other proteins to a hydrophilic interface zone [19].

Unlike globular proteins, amyloid-like structures do not exhibit a monocentric distribution of hydrophibicity. Instead, the local distribution in each unit structure is determined solely by the intrinsic properties of its constituent residues. Environment containing another peptide with a different sequence yet a similar distribution of hydrophobicity results in complexation. Analysis of amyloid fibril structures [31] reveals linear propagation of hydrophobicity peaks interspersed by local troughs, usually along the axis of the fibril.

A "stopper" fragment arrests linear aggregation. A putative drug that exploits this concept would have to fulfill several conditions and be adapted to the specific sequence of its "target" fragment. The proposed drug design model is based on naturally occurring amyloid-like sequences, which have evolved the corresponding "stop" signals preventing unrestricted propagation. The underlying mechanism is discussed in detail elsewhere [22].

### Amphiphilicity and stability of the helix

The proposed helical stopper should, as a rule, be compatible with the local hydrophobicity distribution of the unit structure of the amyloid fibril; however it must also fulfill another condition - propensity for adopting helical, β and random coil conformations for a range of sequences [39]. We used the information contained in the referenced database [39] to select sequences, which exhibit a clear preference for helical forms.

## Results & Discussion

### Identification of amyloid forms

**Figure 1:**
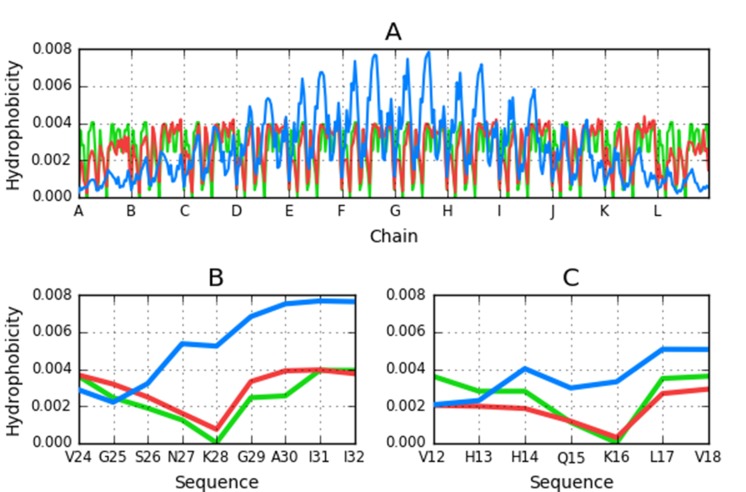
Theoretical (blue) and observed (red) and intrinsic (green) distribution of hydrophobicity in A - the entire complex (2MXU); B - chain F 24-32; C - chain E 12-18.

We show the distribution of hydrophobicity in the 32-residue amyloid β a4 protein (2MXU - 11-42) [31]. As shown in Figure 1, the observed distribution of hydrophobicity is dominated by the intrinsic properties of each residue and does not conform to the monocentric core model as expected by idealized 3D-Gauss distribution of hydrophobicity (blue line on Figure 1.).

Analysis of distribution profiles suggests linear propagation of local hydrophobicity peaks and troughs. This type of structure, devoid of any "stop" signal, would tend to propagate indefinitely. The observed attenuation of hydrophobicity peaks in terminal fragments of the complex and it is caused by the lack of another adjacent peptide. It does not ensure "closure" which would enable the structure to remain water-soluble.

Such linear propagation of two distinct local maxima (Figure 1B & 1C) discordant versus the theoretical one needs to be arrested if the protein is to retain its biological function (comparison of blue line - theoretical distribution - with the observed one - red line - which is highly accordant in respect to intrinsic hydrophobicity - green line). It should be noted that the neighbouring polypeptide chains represent exactly the same hydrophobicity distribution producing the linear propagation.

### A "stop" signal for solenoids:

**Figure 2:**
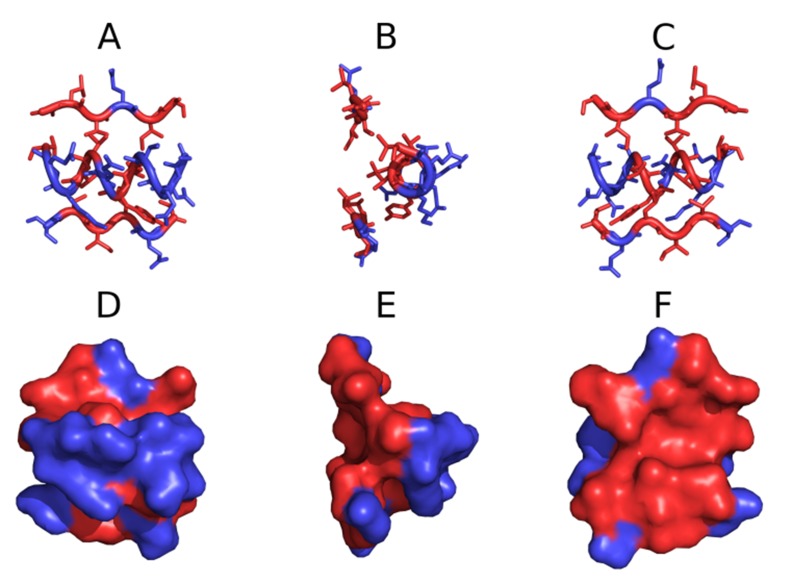
STOP fragment's 3D presentation. Dark blue are hydrophilic residues, red are hydrophobic residues. Top row are transparent all-atom model, bottom row - surface presentation. The structure is viewed from three different perspectives angles: A, D - horizontal orientation of helix seen from the environment site; B, E - helix perpendiculal versus the paper surface, C, F - the "cap" seen from the solenoid perspective.

Solenoids exhibit linear propagation of local hydrophobicity distributions, stretching along their axis. Since such forms are present in naturally occurring proteins, evolution must have come up with a way to counteract their unrestricted propagation. This role falls to a "stop" signal, such as the one present in bacterial chondroitinase b pectate lyase (PDB ID: 1DBG) [31]. We use this structure as a pattern for designing additional polypeptides, which play an identical role with respect to other fibrils. The structure of solenoids is discussed in [22]. Here, we focus on the immediate neighborhood its bracketing "stop" fragments (note that by "neighborhood" we specifically mean the full cyclically occurring structural motif adjacent to each "stop" fragment). It is our understanding that the "stop" fragment must arrest propagation of the linear structure, rendering it capable of interaction with water and thereby counteracting further elongation. Figure 2 presents a graphical depiction of the strongly amphiphilic helix which functions as a "stop" fragment. Since all of its hydrophilic residues face the water environment, no further linear propagation of local hydrophobicity peaks is possible.

### Artificially designed "stop" signals for amyloid peptides

According to the fuzzy oil drop model, linear propagation of fibrils is facilitated by the following phenomena: (1) lack of monocentric hydrophobic core described by a 3D Gaussian; (2) repeatable sequence with alternating hydrophobicity peaks and troughs; (3) distribution of hydrophobicity dominated by the intrinsic properties of each residue; (4) linear propagation of hydrophobicity along a given axis

In order to counteract propagation, the following conditious should be satisfied: (1) the fibri's terminal fragments, along with an adjoining "stop" signal, should be characterized by RD < 0.5, indicating a local distribution of hydrophobicity consistent with the Gaussian model; (2) The "stop" signal should adopt the form of an amphipathic helix; (3) the outward (water-facing) side of the helix should be strongly hydrophilic; (4) the inward (fibril-facing) side should have a distribution of hydrophobicity consistent; with that of the target peptide. We used peptides identified as strongly amyloidogenic as reported elsewhere in this study [23].

### Target peptide of the sequence AIIGLM (PDB ID: 2Y3J):

**Figure 3:**
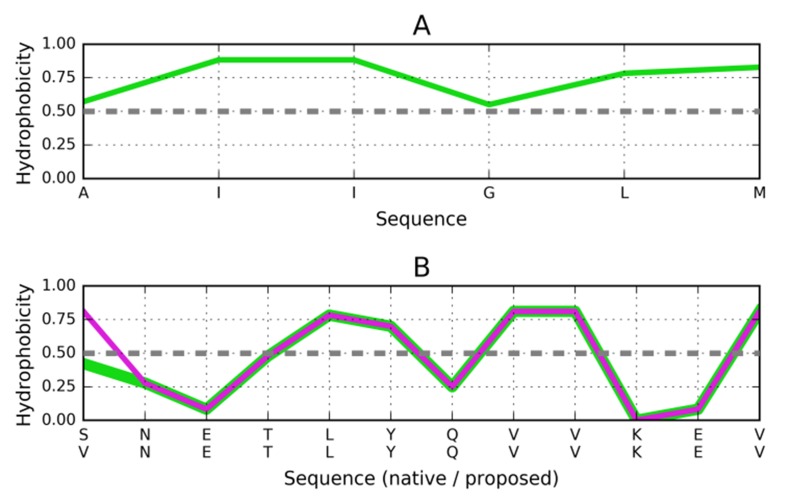
Distribution of hydrophobicity based on the intrinsic properties of residues comprising the AIIGLM (2Y3J). (A) Distribution of hydrophobicity in the target peptide (parallel arrangement). Dashed line separates the hydrophobic (values above 0.5) and hydrophilic (values below 0.5) residues to mach the distribution in the target peptide. (B) Compatible distribution of hydrophobicity in the postulated helix that would attach itself to the fibril. The upper sequence - sequence of the helix as it is present in 1DBG. The lower sequence - proposed as stopper for AIIGLM target peptide sequence. Dashed lines distinguish the high hydrophobicity positions (above 0.5) and low hydrophobicity parameters (below 0.5). The residues with hydrophobicity above 0.5 assumed to interact with target peptide, the residues below 0.5 exposed toward the water environment.

The sequence present in 2Y3J (PDB ID) is characterized by a distribution of hydrophobicity, which closely corresponds to the one in 1DBG (PDB ID). Accordingly, the 1DBG "stop" signal appears to work equally well in 2Y3J. In light of the above, the "stopper" sequence adapted for 2J3Y should be as follows: VNETLYQVVKEV (Figure3). The residues with the hydrophobicity parameter above 0.5 are expected to contact the hydrophobic residues in target peptide, while the residues of hydrophobicity below 0.5 exposed toward the water environment.

### Target peptide of the sequence HSSNNF (PDB ID: 3FPO):

**Figure 4:**
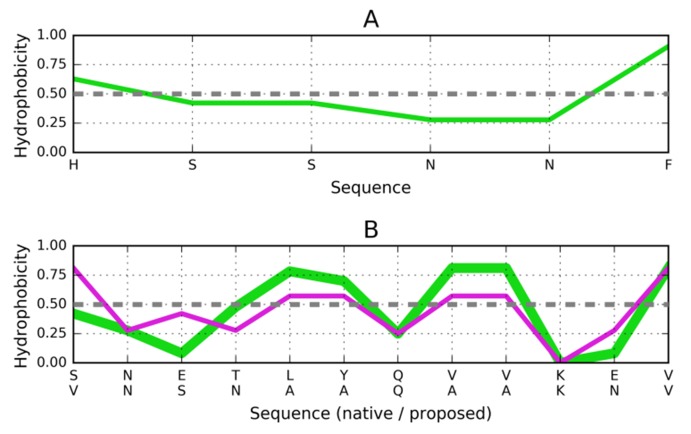
Distribution of hydrophobicity based on the intrinsic properties of residues comprising the HSSNNF fragment (3FPO). (A) Distribution of hydrophobicity in the target peptide (parallel arrangement). (B) Compatible distribution of hydrophobicity in the postulated helix that would attach itself to the fibril. The green line is the hydrophobicity distribution in the pattern helix (1DBG - upper sequence along the X-axis). The magenta line - distribution modified to make the sequence compatible to the target sequence in 3FPO (sequence proposed - lower line below the X-axis). Dashed lines explained in Figure 3.

The HSSNNF sequence is characterized by local maxima on both sides, with relatively low hydrophobicity in the middle. The proposed stopper sequence is VNSNAAQAAKNV (Figure 4). The AAQA and AAKN tetrapeptides tend to adopt helical conformations, as noted in Chseq [39].

### Target peptide of the sequence LSFSKD (PDB ID: 3LOZ):

**Figure 5:**
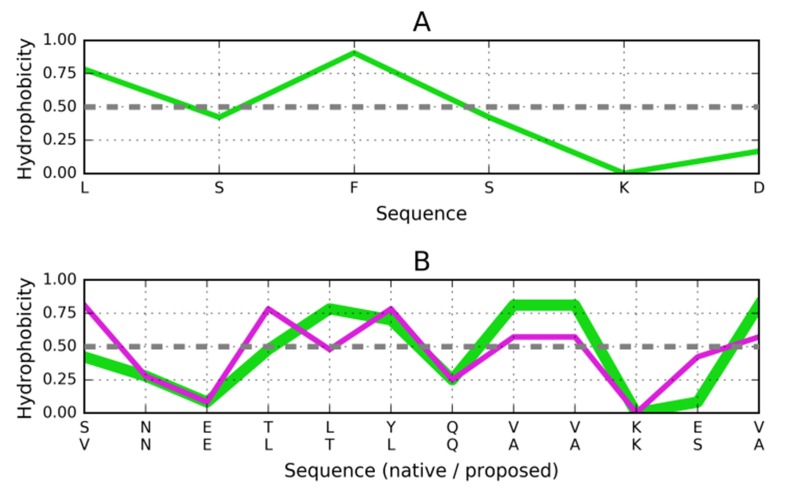
Distribution of hydrophobicity based on the intrinsic properties of residues representing the LSFSKD polypeptide (3LOZ). (A) Distribution of hydrophobicity (parameters) in the target peptide. (B) Compatible distribution of hydrophobicity in the postulated helix, which would attach itself to the fibril. Green line is hydrophobicity distribution in pattern helix (1DBG) (upper sequence below the X-axis), pink line - postulated distribution of hydrophobicity for the helix interacting with target peptide (sequence given in the lower line below the X-axis). Dashed lines explained in Figure 3.

The next target peptide is LSFSKD (3LOZ), with a distribution shown in Figure 5. In this case, we're dealing with a fairly prominent central peak, separated from another distal peak by a shallow trough (residue #2). This information is sufficient to porpose a suitable stopper helix. The proposed sequence is VNELTLQAAKSA (Figure 5), with three strongly helical tetrapeptides (according to Chseq [lit]): ELTL, TLQA and LQAA.

### Target peptide of the sequence MMHFGN (PDB ID: 3NVE):

**Figure 6:**
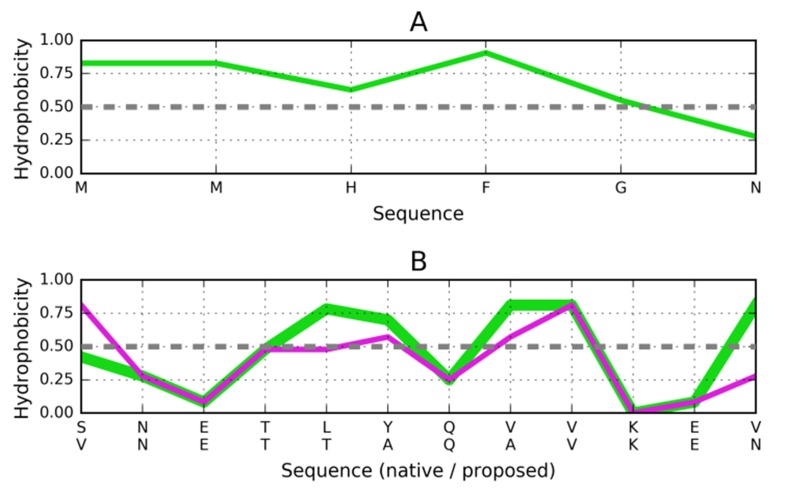
Distribution of hydrophobicity based on the intrinsic properties of residues comprising the MMHFGN fragment (3NVE). (A) Distribution of hydrophobicity in the target peptide (3NVE). (B) Hydrophobicity distribution as postulated for the helix that is expected to attach itself to the fibril. Dashed lines explained in Figure 3.

For the MMHFGN target peptide (3NVE), the matching stopper sequence is VNETTAQAVKEV (Figure 6). This sequence contains four helical tetrapeptides: TTAQ, TAQA, AQAV and AVKE.

### Target peptide of the sequence GYMLGS (PDB ID: 3NHC):

**Figure 7:**
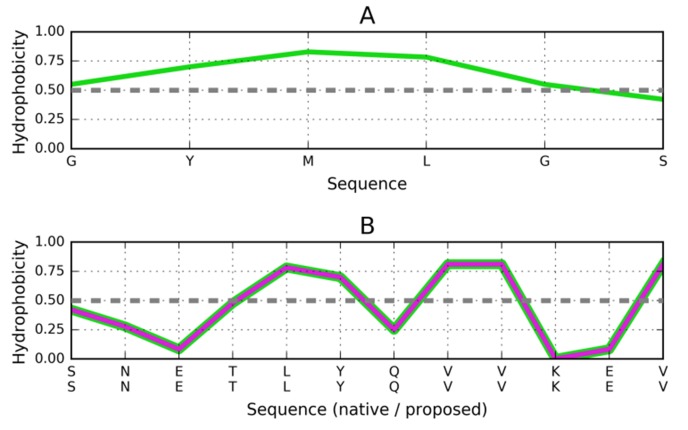
Distribution of hydrophobicity based on the intrinsic properties of residues comprising the GYMLGS fragment (3NHC). (A) Distribution of hydrophobicity in the target peptide. (B) Compatible distribution of hydrophobicity in the postulated helix that would attach itself to the fibril. Dashed line explained in Figure 3.

Here, a similar stoper to the one designed for 1DBG might successfully arrest propagation (Figure 7). The general conclusion regarding "stop" signals is that while dimerization remains a concern, solubility should remain high in all cases.

### Designing stoppers for dual β-fibrils:

**Figure 8:**
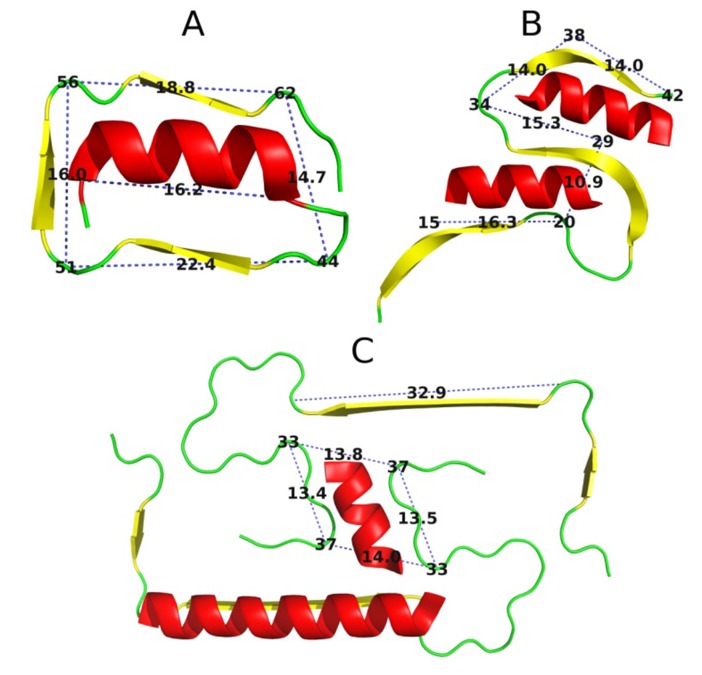
Comparison of separation distances and lengths in target peptides: (A) 1DBG - template used in the design of the proposed stoppers (3D visualization of helix together with the target β-peptides); (B) 2MXU - in this case two potential anchoring points for a "stop" helix are present; (C) 2MVX - here, we focus on two sites: a double β-fragment and a long individual β-fragment; Red - helical fragments expected to be anchored to the β-structural fragments (yellow).

It is interesting to consider potential stoppers adapted to Aβamyloids, such as the ones present in 2MVX and 2MXU. Figure 8 provides a visual depiction of this case, listing the separation and proportions of all fragments, which a suitably designed helix should bracket.

### Proposed helical stopper for 2MVX:

**Figure 9:**
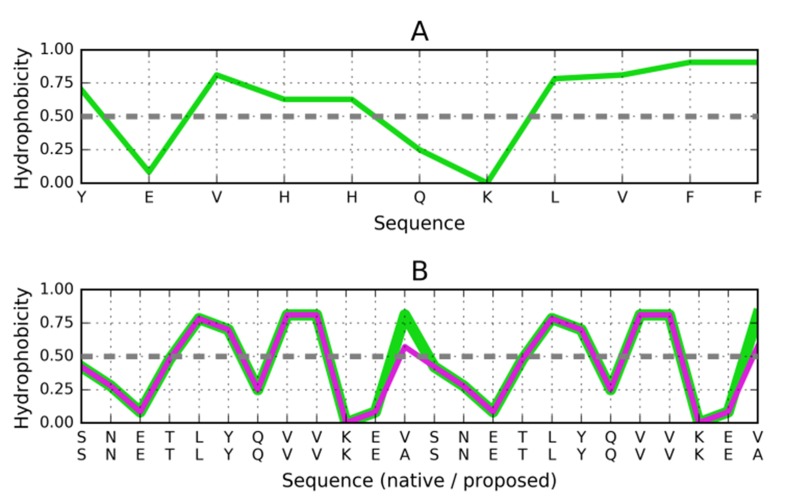
Comparison of hydrophobicity profiles in the proposed "stop" helix and the corresponding β structure in 2MVX (10-20 aa). Red line indicates corresponding residues with compatible hydrophobic interactions. Green line is the pattern sequence as appears in 1DBG. Dashed lines explained in Figure 3.

The proposed sequence for a helix which could potentially obstruct the 10-20 aa section in 2MVX is as follows: SNETLYQVVKE(V)ASNETLYQVVKEA(V). It is a fairly long helix that can span the entire length of the β-fragment. We believe that any shorter fragment of this helix may be sufficient as a stopper to avoid unwanted immune reaction. The sequence is based on the 1DBG template, with a single change marked by square brackets. The introduction of an Ala residue is dictated by the need to reduce hydrophobicity compared to the original Val residue. This substitution has been verified to increase the sequence's affinity for adopting helical conformations. Figure 9 illustrates the local distribution of hydrophobicity in each β- fragment and the corresponding distribution in the proposed helix.

### Proposed helical stopper for (PDB ID: 2MXU):

**Figure 10:**
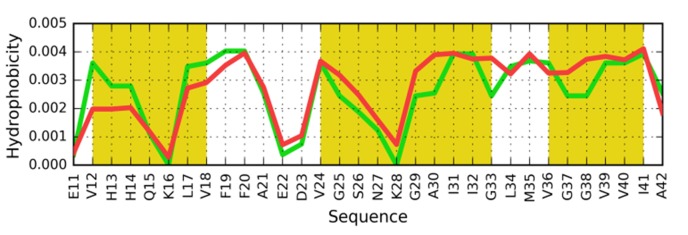
Observed (O - green) and intrinsic (H-red) hydrophobicity distribution profiles for the β-fragments in 2MXU. The yellow fragments are β-fragments.

In this amyloid we can discern two potential anchoring points for a stop signal (Figure 10). The corresponding profile for the proposed helix is illustrated in Figure 10. Designing additional helical stoppers based on the 1DBG template and fulfilling all the previously stated criteria should be relatively trivial for verification.

### Other types of “stop” signals:

**Figure 11:**
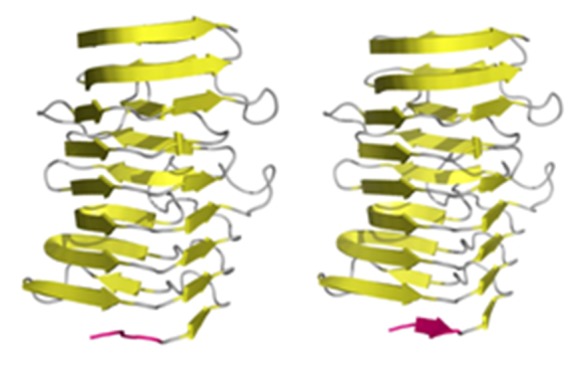
Examples of solenoids terminated by very short β- fragments (red fragments) (C-terminal in 4YZA and the Nterminal fragment in 1DAB).

Our analysis of the terminal sections of linearly ordered fragments revealed some very short bracketing folds, whose length does not exceed half the length of the blocked peptide. It seems that such short β-fragments may also play the role of a "stop" signal are as long as they disrupt the regular ordering of local hydrophobicity peaks, preventing the attachment of another unit peptide (Figure 11).

### Drugs proposed by other authors:

It is difficult to properly discuss the stoppers proposed in [40, 41, 42] since the cited papers lack a clear description of the target peptides. Of note is the high density of polar residues, which might encourage contact with water; however no information regarding the compatibility of the hydrophobic side of the stopper with the target peptide. AEVVFT and TAVVTN [40]. According to the data in [40, 41], the authors assume that the peptide will adopt a β-conformation. This may indeed occur as the peptide aligns itself with the target; however it seems that such peptides may be effective only for certain selected target sequences. The short β-strand highly compatible with respect to β-stand in target fibrillar molecule is shown in 1DAB. This short β-strand should be of 1/3 or even less of the length of the target β-strand.

## Conclusions:

Drug like inhibitors: Peptides with short helix should (1) easily interact with fragments which sustain propagation in fibrils; (2) include hydrophilic elements which enable contact with water and prevent indefinite propagation of linear forms; (3) avoid self-association; (4) exhibit a tendency to form an amphiphatic helical conformation, with the hydrophobic side attached to the fibril (if possible, with high selectivity versus the target molecule of fibril) and the hydrophilic side facing the water environment - such as in 1DBG.

An ampiphatic helix with hydrophobic residues facing the fibril and hydrophilic residues facing the water environment creates a "bridge" between the hydrophobic portion of the fibril and the environment. Note that a helical peptide is typically stable in its isolated form, whereas the stability of an isolated β-strand cannot be ensured. Known peptides discussed elsewhere [40, 41, 42] fulfill some of the conditions specified above. It should also be noted that in order to function as a drug, the peptides should resist dimerization and avoid triggering an immune response.

## Figures and Tables

**Table 1:** Dataset of peptides and proteins used in this study as obtained from elsewehere [23].

Peptide		Sequence	Characteristics	Ref
1YJP	Prion	GNNQQNY	parallel	[24]
2Y3J	Amyloid β	AIIGLM	parallel	[25]
3FPO	Islet amyloid polypeptide	HSSNNF	parallel	[26]
3LOZ	macroglobulin	LSFSKD	antiparallel	[27]
3NVE	prion	MMHFGN	antiparallel	[28]
2Y3K	Amyloid β	MVGGVVIA	antiparallel	[25]
3NHC	Prion	GYMLGS	antiparallel	[29]
2MVX	Amyloid 42aa	Nov-42	amyloid	[30]
2MXU	Amyloid 42aa	Nov-42	amyloid	[31]
1DBG	Lyase		solenoid	[32]
